# Maternal hyperuricemia in normotensive singleton pregnancy, a prenatal finding with continuous perinatal and postnatal effects, a prospective cohort study

**DOI:** 10.1186/1471-2393-14-104

**Published:** 2014-03-18

**Authors:** Elaheh Amini, Mahdi Sheikh, Sedigheh Hantoushzadeh, Mamak Shariat, Alireza Abdollahi, Maryam Kashanian

**Affiliations:** 1 Maternal, Fetal and Neonatal Research Center, Vali-asr Hospital, Tehran University of Medical Sciences, Tehran, Iran; 2 Breastfeeding Research Center, Vali-asr Hospital, Tehran University of Medical Sciences, Tehran, Iran; 3 Department of Pathology, Imam Khomeini Hospital complexes, Tehran University of Medical Sciences, Tehran, Iran; 4 Department of Obstetrics & Gynecology, Akbarabadi Teaching Hospital, Tehran University of Medical Sciences, Tehran, Iran

**Keywords:** Hyperuricemia, Uric acid, Neonatal, Pregnancy, IVH

## Abstract

**Background:**

To assess the association of maternal hyperuricemia with adverse pregnancy outcome and neonatal metabolic, neurologic and respiratory disturbances in normotensive singleton pregnant women.

**Method:**

This prospective multicentric cohort study was conducted on 404 normotensive singleton pregnant women who were admitted for delivery in Vali-Asr and Akbar-Abadi teaching hospitals of Tehran University of Medical Sciences, Tehran, Iran. Upon enrollment maternal and umbilical sera were obtained for determining uric acid levels. 1 and 5 minutes Apgar scores, the need for neonatal resuscitation and neonatal intensive care unit (NICU) admission were recorded. In case of NICU admission a neonatal blood sample was drawn for determining uric acid, blood sugar and bilirubin levels. An intracranial ultrasound imaging was also carried out for the admittd neonates for detecting intraventricular hemorrhage.

**Results:**

Maternal hyperuricemia (uric acid one standard deviation greater than the appropriate gestational age) was independently associated with preterm birth (odds ratio (OR), 3.17; 95% confidence interval (CI), 2.1 – 4.79), small for gestational age delivery (OR, 1.28; 95% CI, 1.04 – 2.57), NICU admission (OR, 1.65; 95% CI, 1.12 – 2.94) and neonatal IVH (OR, 8.14; 95% CI, 1.11 – 87.1).

**Conclusions:**

Maternal hyperuricemia in normotensive singleton pregnant women is significantly associated with preterm and SGA delivery and the development of neonatal IVH.

## Background

Uric acid, the final product of purine degradation, is formed by the liver from endogenous and exogenous precursor proteins and is mainly excreted by the kidneys (65%) and intestines (35%). At physiologic concentrations, uric acid exhibits excellent antioxidant activity; uric acid is responsible for 2/3 of the total plasma antioxidant capacity. However, when uric acid exceeds its physiologic levels in the plasma, it can propagate oxidative damage. Furthermore, chronic, but not acute, elevation of uric acid constitutes a risk factor for many diseases, as it can promote inflammation and endothelial dysfunction [1,2].

Asymptomatic hyperuricemia is a common problem, affecting up to 20% of the general population [3]. In pregnancy, hyperuricemia remains a prevalent problem despite the increase in the glomerular filtration rate (GFR).

Elevated plasma uric acid has been associated with many adverse pregnancy outcomes. Elevated plasma uric acid is an independent risk factor for preterm birth, low birth weight (LBW) delivery and low 1- and 5-minute Apgar scores [4-6]. These findings have led some researchers to recommend screening for hyperuricemia during pregnancy as a useful way to lower adverse pregnancy outcomes [7].

In newborns, increased serum uric acid is also associated with neonatal complications and poor prognosis; Perlman et al. reported that elevated uric acid concentrations in the first postnatal day are associated with the subsequent development of severe intraventricular hemorrhage (IVH) and periventricular leukomalacia (PVL) [8]. In other studies, neonatal hyperuricemia was associated with asphyxia and infant respiratory distress syndrome (RDS) [9,10].

Given that uric acid is freely transferred via the placenta and that neonatal hyperuricemia can be a reflection of maternal hyperuricemia [6], elevated maternal uric acid is postulated to play a role in the pathophysiology of these adverse outcomes; however this remains unproven.

No previous study has assessed the associations and effect of maternal hyperuricemia on short and long-term neonatal complications. Whether maternal hyperuricemia has any associations with neonatal metabolic disturbances remains obscure. Furthermore, the majority of research has focused on the effect of hyperuricemia on pregnancy outcomes in the presence of hypertension and preeclampsia or multiple gestations, all of which can affect pregnancy outcomes and uric acid levels.

We undertook this study to evaluate the effect of maternal hyperuricemia on umbilical and neonatal uric acid levels and pregnancy outcomes, in the absence of any hypertensive disorder or multiple gestations. We also assessed the effect of maternal hyperuricemia on neonatal blood sugar, bilirubin levels and intracranial sonography findings. Further, neonates were followed for one month to see whether maternal hyperuricemia is associated with any long-term neonatal complications.

## Methods

### Study population and study design

This multicentric prospective cohort study was conducted on pregnant women admitted to the Vali-Asr and Akbar-Abadi teaching hospitals of the Tehran University of Medical Sciences, Tehran, Iran, for delivery between September 2011 and June 2012. Pregnant women were considered eligible for the study if they had the following criteria: singleton pregnancy, normal blood pressure upon enrolment, nonsmokers, nonalcoholic, no history of substance abuse, intact fetal membranes, no history of fever or antibiotic use and/or any site of infection in the previous two weeks, no vaginal bleeding upon admission.

A total of 559 pregnant women were asked to participate in the study, and 155 were excluded due to one or more of the following criteria which could affect both serum uric acid levels and pregnancy outcome: any history of smoking; opiate or alcohol consumption during pregnancy; any hypertensive disorder including both pre-existing and gestational related hypertensive disorders; multiple gestations; history of infections, fever or antibiotic use during the previous 2 weeks. After obtaining a written informed consent, a total of 404 women aged 15 – 44 years at gestational ages of 28 – 42 weeks participated in the study, which was approved by the ethics committee of the Tehran University of Medical Sciences.

### Data and specimen collection

Upon enrollment, a standardized questionnaire was completed for every participant through interviews and medical records. The questionnaire contained demographic, medical, gynecological, obstetrical and social history, as well as inquiries about body mass index (BMI) before pregnancy, gestational weight gain and the vital signs obtained by the physical examination. Gestational age was calculated based on ultrasound imaging, and Fenton growth charts were used to calculate birth weight percentiles [11]. Maternal blood samples were drawn within 24 hours preceding delivery to obtain uric acid, creatinine and blood urea nitrogen (BUN) levels. Uric acid levels were determined by the enzymatic colorimetric method, and all laboratory investigations were performed by one person who was blinded to the results of pregnancy outcomes. In the delivery room, after the neonate was delivered, the umbilical cord was double clamped and a blood samples was obtained from the umbilical vein for determining cord blood uric acid levels. Meanwhile the 1- and 5-minutes Apgar scores, the need for neonatal resuscitation, the need for neonatal intensive care unit (NICU) admission and the neonatal sex and weight were recorded. Apgar scores were calculated using the following five criteria: skin color, pulse rate, reflex irritability, muscle tone and breathing. The need for resuscitation was defined as the newborn requiring positive pressure ventilation, chest compression or resuscitative drugs after delivery. The neonates were followed for one month by reviewing their medical records for evidence of respiratory distress syndrome needing surfactant therapy, seizures and pathologic jaundice. Because of ethical issues only in case of NICU admission, a blood sample was drawn during the first 24 hours to assess uric acid, bilirubin and blood sugar levels. Neonatal blood sugar was measured in the first to third postnatal hour. Intracranial ultrasound imaging was performed on all neonates admitted to the NICU during the first 5 postnatal days to detect intraventricular hemorrhage (IVH). The mothers were also followed and examined in the postpartum period for one week for the detection of post partum hypertensive disorders.

### Statistical analysis

All statistical analyses were performed using SPSS statistical software (version 18.0.0: PASW). Chi-squared analysis, Fisher’s exact test, independent-samples *T* test and Pearson correlation coefficient were used to analyze the correlations and relationships between variables. Multivariate logistic regression was used to evaluate the dependency of the obtained results. Sample size was calculated for a power of 90% and an alpha error of 0.05, where a sample size of 103 would be required in each group. Estimated odds ratios (ORs) with 95% confidence intervals (95% CIs) and P value < 0.05 were used to evaluate the statistical significance of the associations and correlations between variables.

## Results

### Descriptive statistics

Five hundred fifty nine pregnant women agreed to participate in the study, 155 of whom were excluded. Of those excluded, 61 had hypertensive disorders, 37 had multiple gestations, 30 had a history of gestational smoking, opiates or alcohol consumption, 27 had infections or antibiotics use during the previous 2 weeks. 404 pregnant women completed the study. At enrollment, the mean ± standard deviation (SD) for maternal age was 28.4 ± 5.7 years; maternal pre pregnancy BMI was 25.5 ± 4.8; gestational weight gain was 12.4 ± 4.7 kg; maternal serum creatinine was 0.7 ± 0.2 mg/dl; maternal BUN was 18.4 ± 7.2 mg/dl; maternal, cord blood and neonatal uric acid were 4.5 ± 1.1, 4.4 ± 1.4 and 5.8 ± 1.7 mg/dl, respectively; gestational age was 38.1 ± 2.2 weeks and neonatal birth weight was 3083.5 ± 611.2 grams.

### The association of maternal hyperuricemia and preterm birth with maternal factors

Seventy two women experienced preterm birth (delivery at gestational age < 37 weeks) (17.8%), which was significantly associated with younger maternal age (P = 0.04), primigravidity (P = 0.02), prior historyof preterm birth (P = 0.01) (Table 1).

**Table 1:** Association of preterm delivery and elevated maternal serum uric acid levels with, obstetrical and medical characteristics of the studied population (n = 404)

**Characteristic**	**Mothers with high vs. normal uric acid values**		**Mothers with preterm vs. term delivery**	
	**PD (n = 72)**	**TD (n =332)**	**HU (n = 103)**	**NUA (n = 301)**
	**N (%)**	**N (%)**	**N (%)**	**N (%)**
Maternal age < 26 years	27 (37.5)	95 (28.6)*	42 (40.7)	80 (26.5)**
Primigravidity	31 (43.1)	114 (34.3)*	44 (42.7)	101 (33.5)*
BMI before pregnancy > 25	35 (48.6)	167 (50.3)	44 (42.7)	153 (50.8)
Gestational weight gain > 14 kg	35 (48.6)	129 (38.8)	42 (40.7)	103 (34.2)*
Prior history of preterm labor	8 (11.1)	11 (3.3)*	7 (6.7)	12 (3.9)
Prior history of miscarriage	20 (27.7)	81 (24.3)	21 (20.3)	81 (26.9)
Gestational anemia	4 (5.5)	19 (5.7)	7 (6.8)	16 (5.3)
Gestational diabetes	7 (9.7)	27 (8.1)	7 (6.8)	27 (9)
Maternal Cr > 1	12 (16.6)	36 (10.8)	22 (21.3)	26 (8.6)**
Maternal BUN > 30 mg/dl	10 (13.8)	28 (8.4)	15 (14.5)	22 (7.3)*

PD: Preterm Delivery, TD: Term Delivery, HU: Hyperuricemia, NUA: Normal Uric Acid levels.

*: P < 0.05 for the comparison between two groups with and without the specified characteristic, **: P < 0.01 for the comparison between two groups with and without the specified characteristic, BMI: Body Mass Index, Cr: serum creatinine (mg/dl), BUN: blood urea nitrogen (mg/dl).

Hyperuricemia was defined as a serum uric acid level one standard deviation greater than the appropriate for gestational age as defined by Lind et al. [12] (Table 2). Use of the gestation corrected uric acid was chosen because the value of uric acid in normal pregnancy is dynamic and fluctuates in a consistent pattern [12], the choice of using “one-standard deviation” above this level is consistent with other published studies and also with standard laboratory protocols to determine “abnormal” values [13]. Hyperuricemia was detected in 103 pregnant women (25.4%) and was significantly associated with younger maternal age (P = 0.005), primigravidity (P = 0.04), excessive gestational weight gain (gestational weight gain > 14 kg) (P = 0.01), elevated maternal serum creatinine (serum creatinine ≥ 1 mg/dl) (P = 0.002) and elevated maternal BUN (BUN ≥ 30 mg/dl) (P = 0.03) (Table 1).

**Table 2:** **Uric acid values that is considered elevated in pregnancy based on gestational age as defined by Lind et al. [**[12]**]**

**Gestational age**	**Serum uric acid (mg/dl)**
28 – 32 W	≥ 4.50
32 W + 1 D - 33 W	≥ 4.70
33 W + 1 D - 34 W	≥ 4.93
34 W + 1 D - 35 W	≥ 4.98
35 W + 1 D - 36 W	≥ 5.04
36 W + 1 D - 37 W	≥ 5.40
38 W and over	≥ 5.58

W: weeks, D: days.

### Pregnancy outcomes overview

Fifty six women gave birth to a small for gestational age (SGA) neonate (birth weight < 10th percentile for gestational age according to Fenton growth charts) [11] (11.3%). Seventy nine neonates required NICU admission (19.5%). Forty neonates had low 1 minute Apgar scores, and 40 neonates had low 5 minute Apgar scores (Apgar score < 7) (9.9%). Twenty seven required resuscitation in the delivery room (6.6%). Seven neonates developed IVH (1.7%) and 16 neonates suffered from RDS (3.9%). Of the admitted neonates from whom blood samples could be obtained, hyperglycemia (blood sugar ≥ 120 mg/dl) was detected in 6 (7.5%), whereas hypoglycemia (blood sugar < 45 mg/dl) was detected in 18 (22.7%). Hyperbilirubinemia during the first 24 hours of life (total bilirubin ≥ 5 mg/dl or conjugated bilirubin ≥ 1.5 mg/dl) was detected in 21 neonates (26.5%).

### Maternal hyperuricemia and its relationship to pregnancy outcomes

By the Pearson correlation coefficient, maternal serum uric acid was significantly correlated with cord blood and first day neonatal serum uric acid (r = 0.41, P = 0.000) and (r = 0.22, P = 0.004), respectively, as well as with maternal serum creatinine (r = 0.18, P = 0.001), and maternal BUN (r = 0.2, P = 0.000).

In Table 3 the association between maternal hyperuricemia and different pregnancy outcome characteristics is illustrated using OR and 95% CI. Maternal hyperuricemia was associated with preterm birth (P = 0.000), SGA delivery (P = 0.02), Low 1- and 5- minute Apgar scores (P = 0.004), NICU admission (P = 0.002), neonatal hypoglycemia (P = 0.009) and neonatal IVH (P = 0.007).

**Table 3:** Association between maternal hyperuricemia and different pregnancy outcome characteristics

**Characteristic**	**Maternal hyperuricemia**		**Unadjusted OR (95% CI)**	**Adjusted OR † (95% CI)**
	**Yes**	**No**		
	**N (%)**	**N (%)**		
SGA	14 (13.5)	32 (10.6)*	1.34 (1.12 – 2.63)	1.28 (1.04 – 2.57)
The need for resuscitation	10 (9.7)	17 (5.6)	1.77 (0.78 – 4.01)	1.27 (0.53 – 3.06)
Low 1-minute APGAR	18 (17.4)	22 (7.3)*	2.64 (1.35 – 5.16)	1.93 (0.94 – 3.99)
Low 5-minute APGAR	18 (17.4)	22 (7.3)*	2.64 (1.35 – 5.16)	1.93 (0.94 – 3.99)
NICU admission	31 (30)	48 (15.9)*	2.26 (1.34 – 3.82)	1.65 (1.12 – 2.94)
Neonatal hyperglycemia	1/31 (3.22)	5/48 (10.4)	0.56 (0.31 – 10.26)	0.32 (0.23 – 7.5)
Neonatal hypoglycemia	10/31 (32.2)	8/48 (16.6)*	3.48 (1.37 – 8.84)	2.45 (0.9 – 6.66)
Neonatal hyperbilirubinemia	7/31 (22.5)	14/48 (29.1)	0.64 (0.27 – 1.48)	0.21 (0.08 – 2.40)
Neonatal RDS	8 (7.76)	8 (2.65)	1.99 (0.88 – 5.07)	1.79 (0.43 – 4.06)
Neonatal IVH	6 (5.8)	1 (0.3)*	12.01 (1.44 – 101.32)	8.14 (1.11 – 87.1)
Neonatal mortality	3 (2.91)	0 (0)	-	-

OR: odds ratio, 95% CI: 95% confidence interval, †: adjusted for gestational age and birth weight, neonatal hypoglycemia was additionally adjusted for maternal diabetes, *: P < 0.05 for the comparison between two groups with and without the specified characteristic, SGA: small for gestational age delivery, APGAR: Apgar score, RDS: respiratory distress syndrome, IVH: intraventricular hemorrhage.

### Dependency of the obtained results

Using multivariate logistic regression, maternal hyperuricemia remained significantly associated with preterm birth (B = 1.15, P = 0.000) when adjusted for younger maternal age, primigravidity and prior history of preterm birth (Table 4). After adjustment for gestational age and birth weight maternal hyperurcemia remained significantly associated with SGA delivery (B = 0.82, P = 0.04), NICU admission (B = 0.5, P = 0.04) and neonatal IVH (B = 0.49, P = 0.03), But not with low 1- and 5- minute Apgar scores (B = 0.66, P = 0.08). When adjusted for gestational age, birth weight and maternal diabetes, maternal hyperuricemia was not significantly associated with neonatal hypoglycemia (B = 0.83, P = 0.07) (Table 3).

**Table 4:** Multivariate logistic regression analysis of factors associated with preterm birth

**Factor**	**B value**	**Unadjusted OR (95% CI)**	**Adjusted OR (95% CI)**	**Adjusted P value**
Maternal age < 26 years	0.2	1.64 (1.1 – 2.44)	1.22 (0.77 – 1.92)	0.38
History of preterm birth	1.4	3.54 (1.61 – 7.77)	4.05 (1.71 – 9.6)	0.001
Primigravidity	0.59	2.05 (1.09 – 3.9)	1.8 (1.15 – 2.82)	0.009
Maternal hyperuricemia	1.15	3.68 (2.46 – 5.49)	3.17 (2.1 – 4.79)	0.000

OR: odds ratio, 95% CI: 95% confidence interval.

## Discussion

### Main findings

Our study showed that there is significant correlation between maternal, umbilical and neonatal uric acid levels and that maternal hyperuricemia is independently associated with preterm birth, SGA delivery, NICU admission and the subsequent development of neonatal IVH.

### Strengths and limitations

The main strengths of this study were the large sample size and evaluating the effect of maternal hyperuricemia on neonatal blood sugar, bilirubin and IVH, which had not been done previously in any study. In addition this study is among the very few studies that searched for the association of maternal hyperuricemia and adverse pregnancy outcome in the absence of any hypertensive disorder.

The main limitation of our study was that uric acid levels where only measured in the third trimester of pregnancy; we have no data on the maternal uric acid concentrations in the first and second trimesters. We do not know whether the mothers exhibited elevated uric acid before gestation or whether uric acid became elevated during pregnancy. The answer to this question is very important and may have clinical implications for any intervention lowering the adverse outcomes associated with hyperuricemia.

Another limitation of the study was that the study was conducted in two referral centers with high percentage of high risk pregnancies; the percentage of poor neonatal outcome in this study was rather high. However by using strict inclusion/exclusion criteria and applying multivariate logistic regression analysis we tried to minimize the selection bias that could affect the study.

### Discussion

In our study hyperuricemia was defined as a serum uric acid level one standard deviation greater than the appropriate for gestational age as defined by Lind et al. [12,13] 25.4% of normotensive pregnant women had elevated serum uric acid levels, which is more than the anticipated prevalence. This shows that our population had higher levels of uric acid than the population studied by Lind et al. [12]. In our study, the mean ± SD of serum uric acid levels among normotensive women in the third trimester of pregnancy was 4.5 ± 1.1 mg/dl, consistent with other studies conducted in the United States, Japan and India [14-16]. However some studies have reported lower rates [17,18]. These differences seem to be related to the socioeconomic and dietary differences in the studied populations.

Maternal factors that were associated with increased maternal serum uric acid were as follows: primigravidity, younger maternal age, excessive weight gain and impaired renal function during pregnancy. These associations have also been identified by other studies [4,14]. The association of excessive weight gain and impaired renal function with hyperuricemia can be explained by the overproduction and reduced renal excretion of uric acid, respectively [14]. The mechanisms by which primigravidity can increase maternal uric acid levels remain obscure. The relationship could be due to the immune maladaptation experienced in the first pregnancy; primiparity represents an immune challenge to the mother because of the semi-allogenic nature of the fetus. The first successful pregnancy can induce adaptive changes that favor immune tolerance in subsequent pregnancies [19]. Another explanation could be less maternal psychosocial adaptation in primigravida [20]. Both of these mechanisms are associated with increased serum uric acid concentrations [21,22].

In this study, maternal hyperuricemia among normotensive pregnant women was associated with SGA and preterm birth. These findings are consistent with other studies conducted in different populations and durations of pregnancy; Laughon et al. studied 212 normotensive pregnant women in Pittsburgh and reported that hyperuricemia during the second trimester or pregnancy was associated with a lower birth weight [23]. In another study of 120 Japanese normotensive pregnant women, Akahori et al. documented that hyperuricemia in the third trimester of pregnancy is an independent risk factor for SGA delivery [4]. These results fulfill two important criteria in evaluating the causal relationship of associated factors: 1) consistency of the associations among studies conducted in different circumstances; and 2) the exposure occurred before the outcome.

Maternal hyperuricemia was associated with an increased likelihood of neonatal NICU admission and lower 1- and 5- minute Apgar scores. These findings demonstrate that newborns of mothers with elevated serum uric acid levels are at increased risk for perinatal distress. Different mechanisms have been proposed for the vicious effect of uric acid on pregnancy outcomes. Uric acid has been shown to elicit a concentration-dependent attenuation of trophoblast invasion and integration into a uterine microvascular endothelial cell monolayer [13]; therefore, uric acid is able to induce the formation of a dysfunctional placenta. Another American study demonstrated the inhibition of placental system A amino acid transport with uric acid, which can cause intrauterine growth restriction [24].

Upon follow–up, the neonates born to mothers with hyperuricemia experienced more hypoglycemia and IVH, and the mortality rate was also elevated in this group. Studies have demonstrated that neonates who suffered from asphyxia, hypoglycemia or IVH exhibit an increased level of uric acid in their sera in comparison with normal neonates [8-10]. Our results regarding the significant association between maternal, umbilical and neonatal serum uric acid levels, supported by studies that demonstrate the free transfer of uric acid through the placenta [6], suggest that the real etiology for these poor neonatal outcomes might be the elevated uric acid in maternal sera; neonatal hyperuricemia may be only a reflection of maternal and umbilical hyperuricemia. This vicious effect of uric acid might be due to the prooxidative effect of uric acid and its ability to promote inflammation and endothelial dysfunction [25,26].

## Conclusion

Maternal hyperuricemia in normotensive singleton pregnant women constitutes a risk factor for adverse pregnancy outcomes and the development of neonatal hypoglycemia and IVH. More studies are needed to confirm these results, as genetic, socioeconomic and dietary factors play key roles in uric acid concentrations. Further, cohort studies are needed to measure serum uric acid before pregnancy and throughout gestation and evaluate the effect of uric acid on pregnancy and neonatal outcomes to determine the practical usefulness of measuring and lowering serum uric acid levels before and throughout pregnancy.

## Competing interests

None of the authors have declared any conflict of interests that may arise from being named as an author on the manuscript.

## Authors’ contributions

EA: study design, carrying out, analysis, interpretation of data and revising the paper. MS: study design, carrying out, analysis, interpretation of data, writing the paper, revising the paper. SH: study design, carrying out, revising the paper. MS: study design, carrying ourt, analysis, revising the paper. AA: study design, carrying out, revising the paper. MK: carrying out, revising the paper. All of the above authors have approved the final version.

## Pre-publication history

The pre-publication history for this paper can be accessed here:

http://www.biomedcentral.com/1471-2393/14/104/prepub
